# Hyperthermia of Magnetically Soft-Soft Core-Shell Ferrite Nanoparticles

**DOI:** 10.3390/ijms232314825

**Published:** 2022-11-26

**Authors:** Venkatesha Narayanaswamy, Jayalakshmi Jagal, Hafsa Khurshid, Imaddin A. Al-Omari, Mohamed Haider, Alexander S. Kamzin, Ihab M. Obaidat, Bashar Issa

**Affiliations:** 1 Research Institute of Medical & Health Sciences, University of Sharjah, Sharjah P.O. Box 27272, United Arab Emirates; 2 Department of Physics, College of Sciences, University of Sharjah, Sharjah P.O. Box 27272, United Arab Emirates; 3 Department of Physics, Sultan Qaboos University, P.O. Box 36, Muscat 123, Oman; 4 Department of Pharmaceutics and Pharmaceutical Technology, College of Pharmacy, University of Sharjah, Sharjah P.O. Box 27272, United Arab Emirates; 5 Ioffe Physical Technical Institute, St. Petersburg 194021, Russia; 6 Department of Physics, United Arab Emirates University, Al-Ain P.O. Box 15551, United Arab Emirates; 7 Department of Medical Diagnostic Imaging, College of Health Sciences, University of Sharjah, Sharjah P.O. Box 27272, United Arab Emirates; 8 Department of Biomedical Engineering, Faculty of Engineering and Natural Sciences, Istinye University, Istanbul 34010, Turkey

**Keywords:** exchange bias, exchange anisotropy, SAR, magnetization, AMF, intracellular hyperthermia

## Abstract

Magnetically soft-soft MnFe_2_O_4_-Fe_3_O_4_ core-shell nanoparticles were synthesized through a seed-mediated method using the organometallic decomposition of metal acetyl acetonates. Two sets of core-shell nanoparticles (S1 and S2) of similar core sizes of 5.0 nm and different shell thicknesses (4.1 nm for S1 and 5.7 nm for S2) were obtained by changing the number of nucleating sites. Magnetic measurements were conducted on the nanoparticles at low and room temperatures to study the shell thickness and temperature dependence of the magnetic properties. Interestingly, both core-shell nanoparticles showed similar saturation magnetization, revealing the ineffective role of the shell thickness. In addition, the coercivity in both samples displayed similar temperature dependencies and magnitudes. Signatures of spin glass (SG) like behavior were observed from the field-cooled temperature-dependent magnetization measurements. It was suggested to be due to interface spin freezing. We observed a slight and non-monotonic temperature-dependent exchange bias in both samples with slightly higher values for S2. The effective magnetic anisotropy constant was calculated to be slightly larger in S2 than that in S1. The magnetothermal efficiency of the chitosan-coated nanoparticles was determined by measuring the specific absorption rate (SAR) under an alternating magnetic field (AMF) at 200–350 G field strengths and frequencies (495.25–167.30 kHz). The S2 nanoparticles displayed larger SAR values than the S1 nanoparticles at all field parameters. A maximum SAR value of 356.5 W/g was obtained for S2 at 495.25 kHz and 350 G for the 1 mg/mL nanoparticle concentration of ferrogel. We attributed this behavior to the larger interface SG regions in S2, which mediated the interaction between the core and shell and thus provided indirect exchange coupling between the core and shell phases. The SAR values of the core-shell nanoparticles roughly agreed with the predictions of the linear response theory. The concentration of the nanoparticles was found to affect heat conversion to a great extent. The in vitro treatment of the MDA-MB-231 human breast cancer cell line and HT-29 human colorectal cancer cell was conducted at selected frequencies and field strengths to evaluate the efficiency of the nanoparticles in killing cancer cells. The cellular cytotoxicity was estimated using flow cytometry and an MTT assay at 0 and 24 h after treatment with the AMF. The cells subjected to a 45 min treatment of the AMF (384.50 kHz and 350 G) showed a remarkable decrease in cell viability. The enhanced SAR values of the core-shell nanoparticles compared to the seeds with the most enhancement in S2 is an indication of the potential for tailoring nanoparticle structures and hence their magnetic properties for effective heat generation.

## 1. Introduction

The coupling of magnetic phases to form a bi-magnetic interface at the nanoscale helps in tuning the magnetic properties of nanoparticles [1,2]. The presence of interface coupling between core and shell phases has shown improved efficiency in permanent magnets, recording media, microwave absorption, and biomedical applications [3,4,5]. Magnetic properties, such as saturation magnetization, exchange bias, and coercivity of coupled bi-magnetic nanoparticles, are greatly influenced by the thickness and nature of the interface phases. The most often investigated systems for exchange bias are with antiferromagnetic/ferromagnetic and antiferromagnetic/ferrimagnetic interfaces [6,7]. The exchange bias effect is predicted to cause improved efficiency in magnetothermal conversion due to the magnetic interface effect caused by the individual phases [8,9,10]. There are a significant number of reports in which the geometry of a nanoparticle is manipulated to tune the relaxation process to achieve high magnetic heat conversion efficiency [11]. Extensive research was conducted on the use of precisely engineered magnetic nanoparticles for applications, including MRI contrast agents, drug delivery carriers, tissue engineering scaffolds, performance-enhancing therapeutic agents, and even in SARS-CoV-2 detection strips [12,13,14].

Magnetic nanoparticles, when subjected to an alternating magnetic field (AMF), act as nano-heaters. Tumor cells have compact vascular structures, which renders heat dissipation and thermoregulation more difficult. Therefore, hyperthermia causes cells to undergo apoptosis in direct response to applied heat, while healthy tissue maintains the normal temperature [15,16]. Elevated oxidative stress because of increased metabolism is another reason for cell death [17]. The survival times of tumor cells heated above 42 °C for 30–40 min are reduced, leading to death. Nanoparticle cellular uptake and the mechanism of cell death due to hyperthermia are of paramount importance in explaining their eventual clinical applications [18,19]. Mamani et al. reported an in vitro study that evaluated the magnetic hyperthermia technique for the internalization of amino saline functionalized ferrite nanoparticles to target glioblastoma tumor cells in static and dynamic magnetic fields [20]. They were able to achieve up to 70% cell death when exposed for 40 min to an AMF (with a frequency of 557 kHz and an intensity of 300 Gauss). To treat any cancer tumor using magnetic nanoparticles, efficient magnetic heat conversion is necessary to reduce the dose of nanoparticles. The efficiency of superparamagnetic nanoparticles for magnetic hyperthermia depends on the Neel and Brown relaxation processes [21]. Neel relaxation occurs with the rotation of a magnetic moment, whereas Brownian relaxation occurs through the actual rotation of the particles under an AMF. These relaxation processes depend on several factors, such as saturation magnetization, magnetic anisotropy, the viscosity of the local medium, particle size, and the shape of the nanoparticles. The heating efficiency of nanoparticles is expressed in terms of the specific absorption rate (SAR), which is the rate of power absorption per unit mass of the tissues. The linear response theory (LRT) is a widely accepted framework to analyze the power absorption of magnetic nanoparticles for magnetic hyperthermia at low magnetic fields [22]. In the LRT, the SAR, expressed in watts per gram, is provided by [23]:

(1) $$\mathrm{SAR}\left(f,H\right)=\frac{{\pi \mu }_{0}{\;\chi^{\prime \prime }\;H}^{2}f}{\rho }$$

Here, ρ is the mass density of the magnetic material, µ_0_ is the vacuum magnetic permeability, f is the frequency, H is the amplitude of the AMF, and $\chi^{\prime \prime }$, the imaginary part of the magnetic susceptibility, is provided with $\chi =\chi^{\prime }-i\chi^{\prime \prime }$. In LRT, it is assumed that χ stays constant as H increases. It is known that this assumption is valid for very small values of H. Thus, in the LRT, the heat dissipation of the MNPs has a linear dependence on the AMF frequency and a quadratic dependence on the AMF amplitude. The detailed mathematical expressions are provided in the supplementary data. To achieve high heating efficiency for the nanoparticles, it is important to tune these properties by employing suitable synthetic procedures.

This study aims to examine the effect of a soft-soft interface in the ferrite core-shell system of nanoparticles on the magnetic properties and magnetothermal effect. The nanoparticles were synthesized via the well-reported organometallic decomposition of organic salts in high-boiling solvents [24]. The potential to combine the unique features of different magnetic phases (hard-soft and soft-hard) to fabricate nanostructures more effectively has gained attention in core-shell geometry for magnetic hyperthermia applications. Recent studies have demonstrated that bi-magnetic core-shell nanostructures could increase magnetic nanoparticle efficiencies in various applications. Additionally, the presence of exchange-coupled interactions has an impact on the spin reversal processes, and the magnetic characteristics of the materials in the core and shell affect the magnetothermic effect [25,26,27]. The core-shell nanoparticle size distribution and shape are controlled by varying the initial concentrations of the nanoparticles. The shell thickness-dependent magnetic properties at low and room temperatures were studied to determine their effects on the magnetothermal properties. The hyperthermia efficiency was determined for nanoparticles in an agar gel medium. These parameters are important for understanding the local temperature fluctuations, as the local distribution of the nanoparticles would be inhomogeneous after injection into the tumor site. Furthermore, the efficiency for the in vitro cell death of the human breast cancer cell line MDA-MB-231 and human colorectal cancer cell HT-29 cells was studied using the S2 core-shell nanoparticle in an AMF (with a 384.5 kHz frequency and a 350 G field strength). We optimized the time of the AC field exposure required to obtain high toxicity for killing the cancer cells. The cell death mechanism was examined using the flow cytometry method. The contribution of the magnetic field alone for cell killing was studied by employing similar conditions.

## 2. Results and Discussion

### 2.1. Structural and Magnetic Characterization of the MnFe_2_O_4_ Nanoparticle Seeds

**Figure 1:**
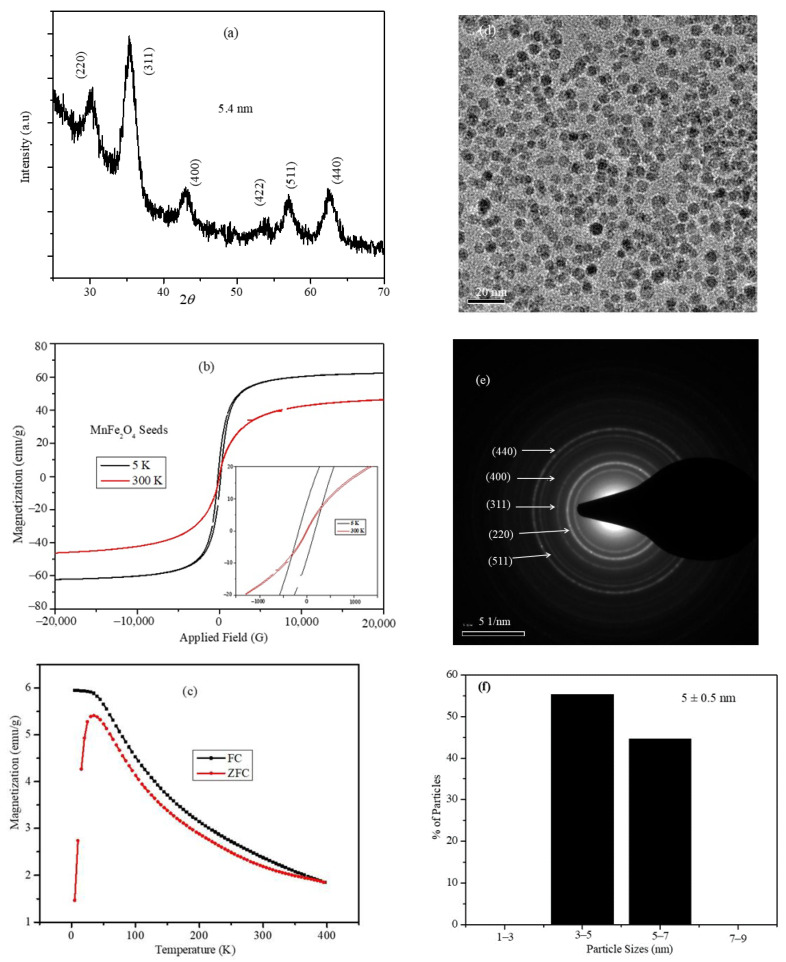
(**a**) XRD pattern, (**b**) field-dependent magnetization hysteresis loops at 5 and 300 K, (**c**) ZFC and FC temperature-dependent magnetization plots, (**d**) bright-field TEM image, (**e**) SAED pattern, and (**f**) size distribution of the MnFe_2_O_4_ nanoparticles.

The XRD profile of the MnFe_2_O_4_ nanoparticles is shown in Figure 1a; the diffraction peaks are indexed to the cubic spinel structure of the MnFe_2_O_4_ nanoparticles with the space group Fd3m (JCPDS card No. 75-0034) [28]. The average particle size of the nanoparticles was calculated using the Scherrer equation and the FWHM of the highest intensity peak (311). The average size of the nanoparticles was 5.4 nm and the lattice constant determined from the multiple-peak fitting of the XRD peaks was 8.4121 Å, which matches the reported values of the MnFe_2_O_4_ nanoparticles of a similar size [29]. The magnetization vs. the applied field was measured at room temperature (300 K) and at 5 K, and the plots are shown in Figure 1b. The MH plots of the nanoparticles show the superparamagnetic nature of the MnFe_2_O_4_ nanoparticles at 300 K. The MH curve at 5 K shows a loop opening, a significant coercive filed (171.2 Oe), and remnant magnetization (8.82 emu/g) as shown in the inset of Figure 1b. The ZFC and FC plots of the nanoparticles were measured by applying a 100 Oe field while cooling (Figure 1c). The blocking temperature was 34.3 K, which corresponds to the peak in the ZFC plot above, in which the particles are superparamagnetic in nature. The bright-field TEM image, SAED pattern, and size distribution histograms obtained by measuring the size of more than 200 individual nanoparticles are shown in Figure 1d–f, respectively. The XRD and SAED patterns indicate that the nanoparticles contained only the ferrite phase and that the lattice parameters agree with the reported values of the MnFe_2_O_4_ nanoparticles. The nanoparticles are spherical in shape and exhibit monodisperse size distribution with an average size of 5.0 ± 0.5 nm.

### 2.2. Structural and Magnetic Characterization of the MnFe_2_O_4_-Fe_3_O_4_ Core-Shell Nanoparticles

**Figure 2:**
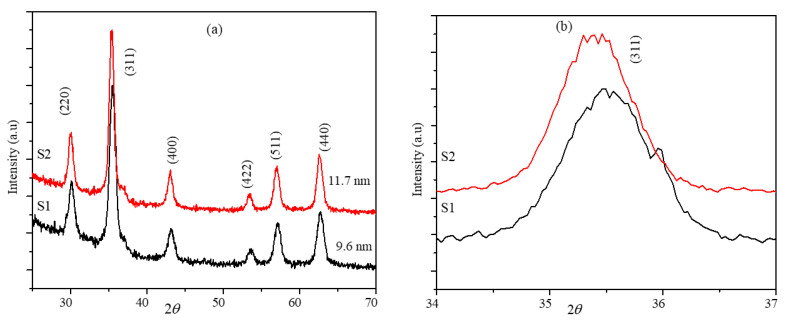
(**a**) XRD of the core-shell nanoparticles S1 and S2. (**b**) Shift in the highest intensity peak (311) of the S1 and S2 nanoparticles.

The XRD diffraction patterns of the core-shell nanoparticles S1 and S2 are shown in Figure 2a. The core-shell nanoparticles show peaks corresponding to the ferrite phase and did not show any additional peaks for Fe_2_O_3_ [28]. The peaks of both core-shell nanoparticles are indexed for the ferrite phase peaks (220), (311), (400), (422), (511), and (440). The average crystalline sizes of the core-shell nanoparticles determined from the FWHM of the (311) peak using the Scherrer equation are 9.6 and 11.7 nm for particles S1 and S2, respectively. The XRD peaks of the S2 nanoparticles are shifted to the lower diffraction angle compared to the peak positions of the S1 nanoparticles (around 35°), as shown in Figure 2b. This shift in the peak position is attributed to the difference in the phase fractions of the MnFe_2_O_4_ and Fe_3_O_4_ ferrites [30]. The lattice parameters of the seed and core-shell nanoparticles were determined using the unit cell program, which uses a non-linear least squares method and regression. The lattice constants of the core-shell nanoparticles are 8.3844 and 8.4010 Å for S1 and S2, respectively. The lattice constants of the seed and core-shell nanoparticles are comparable with the reported values of MnFe_2_O_4_ and Fe_3_O_4_ individual nanoparticles [31]. The lattice parameters of the nanoparticle systems presented here are compared in Table 1.

**Figure 3:**
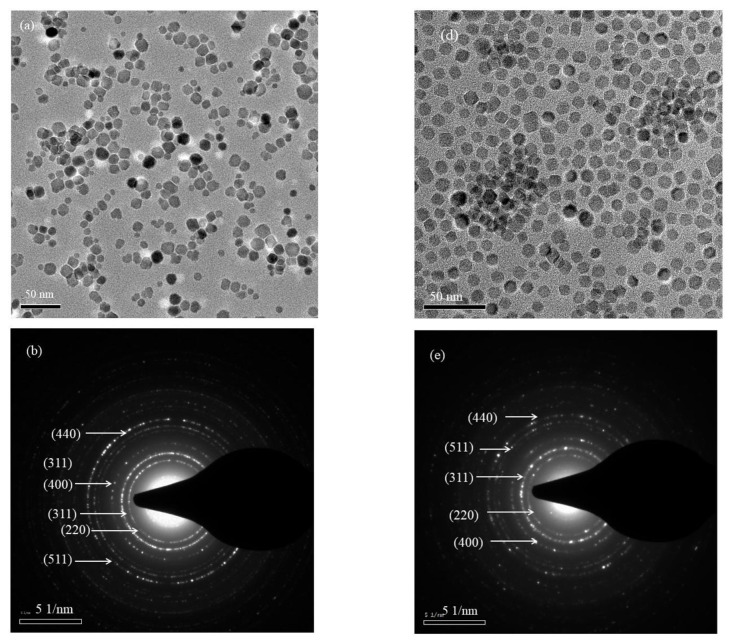

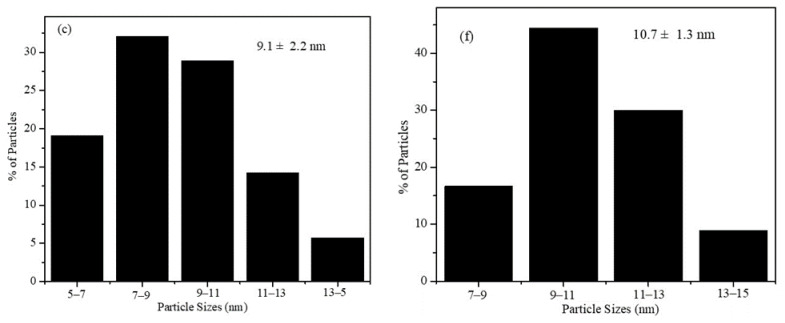
TEM bright-field images, SAED patterns, and size-distribution histograms of the S1 (**a**–**c**) and S2 (**d**–**f**) core-shell nanoparticles.

The bright-field TEM images, SAED patterns, and size-distribution histograms of the S1 and S2 nanoparticles are shown in Figure 3a–f. The TEM images of both sets of core-shell nanoparticles show irregular non-spherical shapes for most of the particles with multiple edges. The S1 nanoparticles have a broad size distribution compared to the S2 nanoparticles. The average sizes of the nanoparticles obtained by measuring the size of more than 200 individual nanoparticles are 9.1 ± 2.2 and 10.7 ± 1.3 nm for the S1 and S2 nanoparticles, respectively. While measuring the size of the individual particles, a good amount of care was taken to avoid overlapped particles, and only the particles with a well-defined outer boundary were considered. The average sizes obtained from the TEM images are in good agreement with the average crystalline sizes calculated from the XRD. The shell thicknesses were obtained by subtracting the average size of the seed nanoparticles from that of the core-shell nanoparticles (obtained from the TEM). This resulted in shell thicknesses of the S1 and S2 nanoparticles of 4.1 and 5.7 nm, respectively. The S1 nanoparticles showed a broad size distribution compared to the S2 nanoparticles, which is attributed to the initial conditions used for the synthesis. The initial nucleation and growth of the Fe_3_O_4_ phase depended on the availability of the MnFe_2_O_4_ surface for the iron precursors. The number of seed particles used for preparing the S1 particles was smaller than for the S2 nanoparticles (40 mg of 60 mg). This produced fewer surface sites and a random growth rate of the Fe_3_O_4_ phases. This shows that 60 mg of seed nanoparticles provided conditions that led to the controlled nucleation and growth of the shell phase, which led to a narrow size distribution. The diffraction patterns of both core-shell nanoparticles agree with the reported patterns of ferrite nanoparticles, which indicates the absence of any other oxide phases [29].

The elements (Mn, Fe) in the ferrite core and shell phases presented here have similar atomic numbers, and thus there are no visible differences in the contrasts of the bright-field TEM images to identify the core and shell boundaries. Hence, confirming the core-shell structure of our samples was a major obstacle. To address this issue, we compared the results with several reports of ferrite core-shell nanoparticle systems using an organometallic decomposition method for the synthesis of core-shell nanoparticles [32,33]. These reports suggested that in the seed-mediated method, the surface of the nanoparticles (MnFe_2_O_4_) would act as a nucleating site for the nucleation and subsequent growth of the shell phase (Fe_3_O_4_), which is thermodynamically favorable rather than phase separation. This gives support to our claims of the core-shell structure since we used a similar synthesis method.

**Figure 4:**
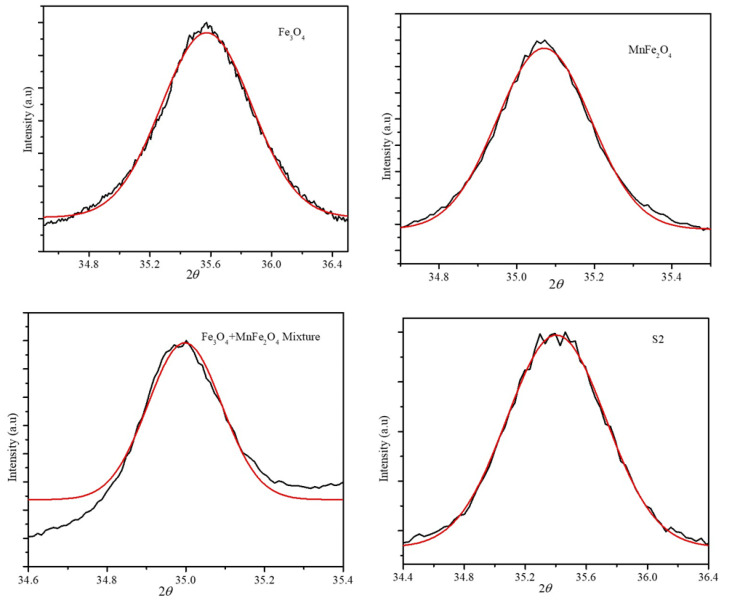
The (311) peak positions obtained from the Gaussian fitting of the XRD diffraction peak (Black-Experimental data and Red-Fitted data).

In addition, the core-shell structure of the nanoparticles was confirmed from the peak positions of the (311) XRD peak of the Fe_3_O_4_ and MnFe_2_O_4_ nanoparticles, and the mechanical mixture of the Fe_3_O_4_ and MnFe_2_O_4_ nanoparticles (with a 1:1 weight ratio) [34]. These peak positions were compared with the peak positions obtained from the S1 and S2 synthesized nanoparticles. The (311) peak position was obtained in the angle range of 34–36° with a step size of 0.01 and exposure time of 100 s/point. The positions were obtained from the Gaussian fitting of the XRD peak-fitted curves, as shown in Figure 4. The peak positions of the individual phases, mechanical mixture, and core-shell nanoparticles is listed in Table 2. The XRD (311) peak position of the mechanical mixture was at 35.0461°, which indicates an overlapping of the (311) peaks of the individual nanoparticle phases. Interestingly, the (311) peak positions of the S1 and S2 core-shell nanoparticles were lower than those of the individual phases. This could be due to the epitaxial growth of the shell phase, which resulted in a strain at the interface caused by the core-shell geometry, which resulted in a significant shift in the peak position.

### 2.3. Magnetic Measurements of the MnFe_2_O_4_-Fe_3_O_4_ Core-Shell Nanoparticles

**Figure 5:**
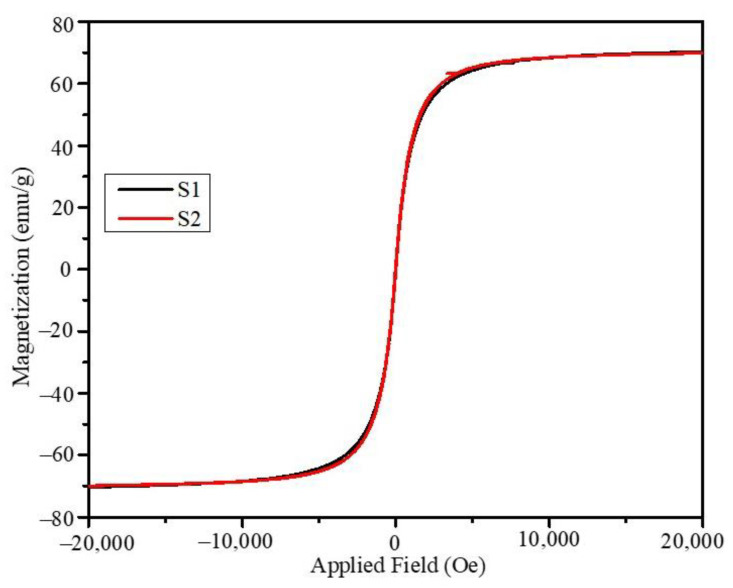
MH plots of the S1 and S2 core-shell nanoparticles at 300 K.

Magnetic hysteresis (MH) loops were obtained under 0, 1, 2, and 3 T field cooling at temperatures of 25, 50, 100, 200, and 300 K. The room temperature superparamagnetic nature of the core-shell nanoparticles is the essential property necessary for the magnetic nanoparticle to be used for biomedical applications. The MH plots obtained at 300 K for the S1 and S2 nanoparticles under zero-field cooling are shown in Figure 5; the saturation magnetization of the nanoparticles shows similar variations in magnitude with respect to the applied fields. The saturation magnetization was nearly 70 emu/g for both the S1 and S2 samples which is lower compared to the individual ferrite bulk phases (Fe_3_O_4_-90 emu/g and MnFe_2_O_4_-112 emu/g) [35,36]. The hysteresis loops obtained under 1, 2, and 3 T field cooling are provided in the supplementary data (Figure S1a–d for S1 and Figure S1e–g for S2). The hysteresis loops showed significant openings at low temperatures (below 100 K), whereas at room temperature, the particles were superparamagnetic with negligible remnant magnetization and coercivity. We observed an opposite trend in our study reported recently for CoFe_2_O_4_-Fe_3_O_4_ hard-soft interface nanoparticles of comparable sizes, which were synthesized using an organometallic decomposition method under similar synthesis conditions of reflux temperatures and concentrations of precursors and solvents [37]. Interestingly, the soft-hard interface nanoparticles showed a significant effect of shell thickness on the saturation magnetization. The saturation magnetization of the different core-shell geometries and comparable sizes are listed in Table 3. The soft-soft interface core-shell nanoparticles do not show any effect on the saturation magnetization.

**Figure 6:**
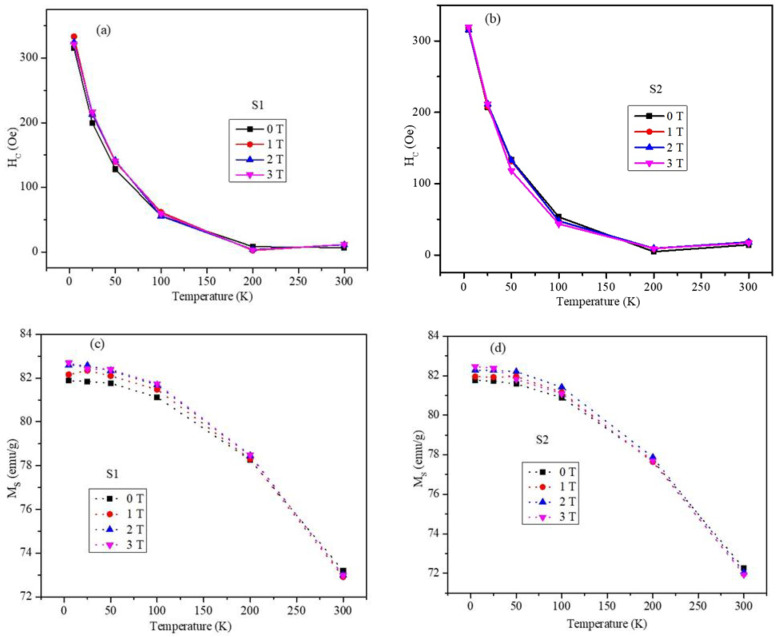
Coercivity as a function of temperature and field cooling for the 0, 1, 2, and 3 T field-cooling values for the (**a**) S1 and (**b**) S2 nanoparticles. Saturation magnetization as a function of temperature and field cooling for the 0, 1, 2, and 3 T field-cooling values for the (**c**) S1 and (**d**) S2 nanoparticles.

The temperature-dependent coercivity and saturation magnetization values were deduced from the MH plots. The temperature and field cooling-dependent coercive fields are shown in Figure 6a S1 and Figure 6b S2. Both sets of nanoparticles displayed similar trends and comparable magnitudes of coercivity in the temperature range of 5–300 K. The nanoparticles possessed a high coercivity of 315 Oe at 5 K, which is higher than that in the seed MnFe_2_O_4_ nanoparticles (H_C_ = 171.2 Oe). The coercivity of the nanoparticles decreased sharply from 5 K and remained constant from 200 to 300 K. The negligible values of the coercivity values at 300 K indicated that the core-shell nanoparticles were superparamagnetic at room temperature, which is essential for the biological applications of nanoparticles. The saturation magnetization (M_S_) of the core-shell nanoparticles obtained at 2 T from the FC MH plots for both samples are shown in Figure 6c,d for the field-cooled values of 0, 1, 2, and 3 T. The magnitudes and temperature dependencies of the saturation magnetizations for both samples were very similar. The saturation magnetization values decreased gradually from 5 to 100 K and dropped sharply above 100 K.

**Figure 7:**
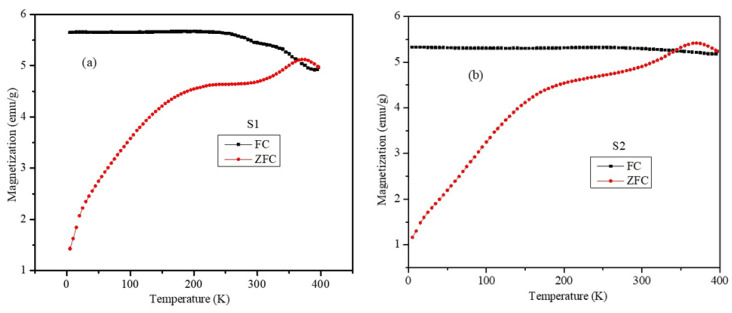
The M-T plots under the ZFC and FC conditions for the (**a**) S1 and (**b**) S2 samples.

The temperature dependence magnetization (M-T) plots under the ZFC and FC conditions at 100 Oe were obtained for the S1 and S2 nanoparticles and are shown in Figure 7. The ZFC plots of both particles showed a broad peak around 230 K and another well-defined peak at approximately 370 K. The soft peak at 230 K is due to the spin glass behavior of the soft-soft ferrite phase interface, which arises from the seed-mediated growth of the Fe_3_O_4_ phase over the MnFe_2_O_4_ core with different lattice parameters. The well-defined peak at 372 k is due to the ferrimagnetic nature of the nanoparticles. The nature of the ZFC and FC plots of the core-shell nanoparticles is significantly different compared to the single-phase MnFe_2_O_4_ nanoparticles, which is due to the broad size distribution and magnetic interface of the MnFe_2_O_4_-Fe_3_O_4_ ferrite phases. The flattening of the FC magnetization is suggested to be a signature of the spin glass (SG) regions that occurred at the core-shell interface of the nanoparticles [38]. We noticed that the flattening of magnetization occurred at temperatures below 250 K in the S1 sample and below 300 K in the S2 sample. We suggest that this is an indication of the existence of larger interface SG regions in the S2 sample than those in the S1 sample. This could hint at larger interface SG regions in samples with larger shell thicknesses.

**Figure 8:**
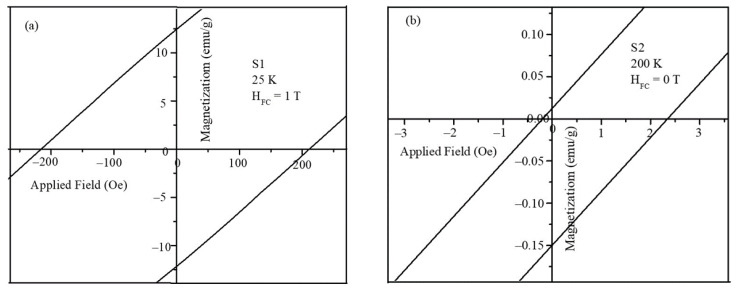
Zoomed portion of the MH plots used for the exchange bias calculations (**a**) S1 at 25 K under 1 T and (**b**) S2 at 200 K under 0 T.

The exchange bias values of the core-shell nanoparticles were obtained from the hysteresis loops of Figure S1a–h in the supplementary data. To calculate the exchange bias values and coercivity of the nanoparticles, the shift in the MH loops at the origin under different field-cooling values was used. The zoomed portion of the MH plots is shown in Figure 8a,b, from which the shift in the magnetization and field values were obtained.

The temperature-dependent exchange bias field showed non-monotonic dependence in both samples (as shown in the supplementary data in Figure S2a,b). We believe that the non-monotonic behavior of the exchange bias field with temperature and field is a signature of the existence of SG regions at the core-shell interface of the nanoparticles. Above 200 K, the exchange bias field values are negligible in both samples. The exchange bias values showed slight dependency on the cooling field in both samples. Because the exchange coupling between the core phase and all interface SG regions is antiferromagnetic (ferromagnetic), a positive (negative) exchange bias appears [6,37,39]. The magnetic field and temperature both influence the exchange coupling in an unpredictable manner. This is because the magnetic coupling between the interface SG regions and the core and shell spins are affected by temperature and the field in an unpredictable manner. In addition, exchange couplings exist among the interface SG regions, which also vary with the field and temperature. A large magnitude of the exchange bias field is a result of a strong net indirect interfacial exchange bias. As expected, the interfacial exchange coupling strength diminished with increasing temperature. The magnitude of the exchange bias field was larger in the S2 sample, thus hinting at larger interface SG regions in the S2 sample than those in the S1 sample.

**Figure 9:**
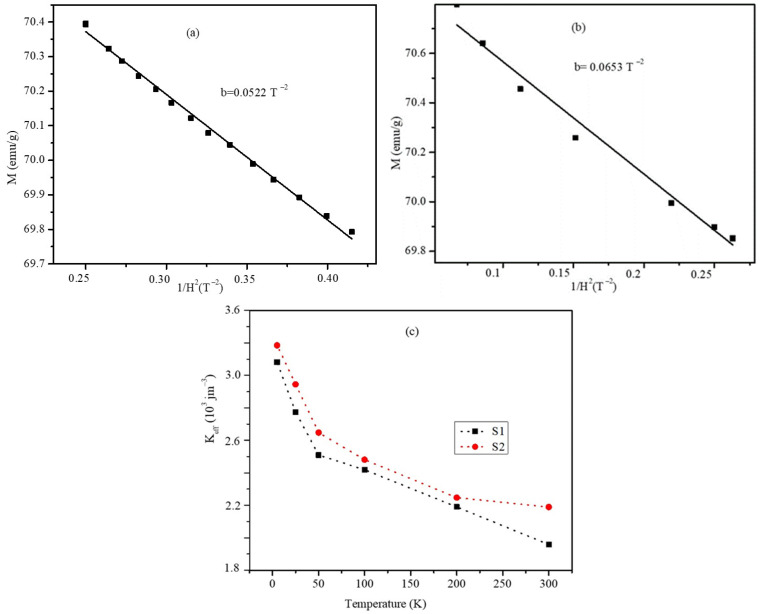
M vs. 1/H^2^ plots obtained at high field strengths for the (**a**) S1 and (**b**) S2 nanoparticles at room temperature (experimental data are marked by symbols, and the solid lines represent a linear fit of the experimental data using Equation (4)). (**c**) Temperature-dependent K_eff_ values of the S1 and S2 nanoparticles.

The effective magnetic anisotropy values and, thus, the magnetic hyperthermia of the nanoparticles would be affected by the presence of the interface SG regions, which results in indirect interfacial exchange coupling. The law of approach to saturation (LAS), which defines the dependency of the magnetization (M) on the applied magnetic field (H) at high field strengths, was used to calculate the effective anisotropy constant (K_eff_) of the particles [40]. According to the LAS, the magnetization near saturation is represented using Equation (4). To calculate the K_eff_ values, the experimental curves of M as a function of 1/H2 were fitted at high magnetic field strengths to obtain the M_S_ and the fitting parameter b, which is associated with K_eff_, as provided in Equation (5). It can be seen from Figure 9c that K_eff_ decreases non-linearly with increasing temperature. However, the K_eff_ values in the S2 nanoparticles were always larger than those of the S1 nanoparticles at the same temperature.

(2) $$M=M_{s}\left[1-\frac{b}{H^{2}}\right]$$

where the parameter b is associated with Keff provided in the following equation:

(3) $$K_{\mathrm{eff}}=\mu_{0}M_{s}\sqrt{\frac{15b}{4}}$$

### 2.4. Magnetic Hyperthermia Studies of the Core-Shell Nanoparticles in Phantom Agar Gel 

The nanoparticles synthesized using the organometallic decomposition method were coated with oleic acid and were not dispersible in water. The nanoparticles were coated with a biocompatible chitosan polymer via a ligand exchange reaction using an optimum amount of polar (DMSO (dimethyl sulfoxide)) and non-polar (hexane) solvents [24]. A total of 100 mg of core-shell nanoparticles were dispersed in 50 mL hexane, and this dispersion was mixed with DMSA (2,3-dimercaptosuccinic acid) into a DMSO (dimethyl sulfoxide) solution with a 10% (w/v) ratio. This solution mixture was sonicated for 30 min; during this process, the nanoparticles were moved to the DMSO phase. The DMSA-coated particles were separated using an external magnet and washed several times using water. These particles were further dispersed in distilled water, and 20 mg of chitosan dissolved in acetic acid was added to this solution along with 20 mg of 1-ethyl-3-carbodiimide hydrochloride (EDC) and left for 24 h. The chitosan was linked to the surface of the nanoparticles through the glycosidic linkage of amide bonds assisted with the EDC and finally washed with water a few times before dispersing in water.

Hyperthermia measurements were carried out in agar gel medium using frequencies of 495.25, 384.5, 347.55, 330.55, 304.75, and 167.30 kHz and field strengths of 200–350 G. A total of 1, 2, and 3 mg/mL of chitosan-coated nanoparticles were dispersed in 1 % agar water medium and heated using a microwave oven to dissolve the agar, and then cooled to room temperature to form a homogeneous ferrogel. Heating profiles were measured for the ferrogel to reach a temperature above 42 °C (measured using an optical temperature probe).

**Figure 10:**
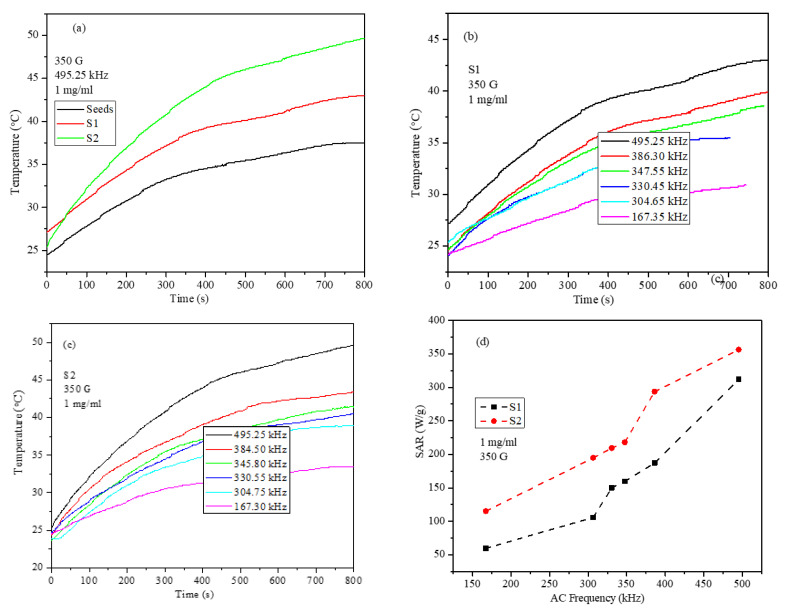
(**a**) Heating profiles of the seed, S1, and S2 core-shell nanoparticles at 495.25 kHz and a 350 G field strength, (**b**) frequency-dependent heating profiles of the S1 (1 mg/mL) nanoparticles under 350 G, (**c**) frequency-dependent heating profiles of the S2 (1 mg/mL) nanoparticles under 350 G, and (**d**) frequency-dependent SAR values of the S1 and S2 nanoparticles under a 350 G AC field in the frequency range of 495.25–167.30 kHz.

A comparison between the heating profiles obtained at a frequency of 495.25 kHz and 350 G for the seed, S1, and S2 core-shell nanoparticle ferrogels is shown in Figure 10a–c. The heating curves clearly show that the S2 nanoparticles possess high magnetothermal conversion under a certain AMF frequency and field. The SAR values of the core-shell nanoparticles obtained at 350 G and various frequencies are shown in Figure 10d. The seed, S1, and S2 nanoparticles’ SAR values were 150.6, 311.8, and 356.5 W/g, respectively, at 495.25 kHz and 350 G. Both S1 and S2 nanoparticles showed similar trends with the AC frequency; however, the SAR values for the S2 sample was higher than those for the S1 nanoparticles at all frequencies. The concentration, AC field, and frequency-dependent heating efficiency of the S2 nanoparticles were studied in detail, and the in vitro examination of killing the cancer cells was studied.

**Figure 11:**
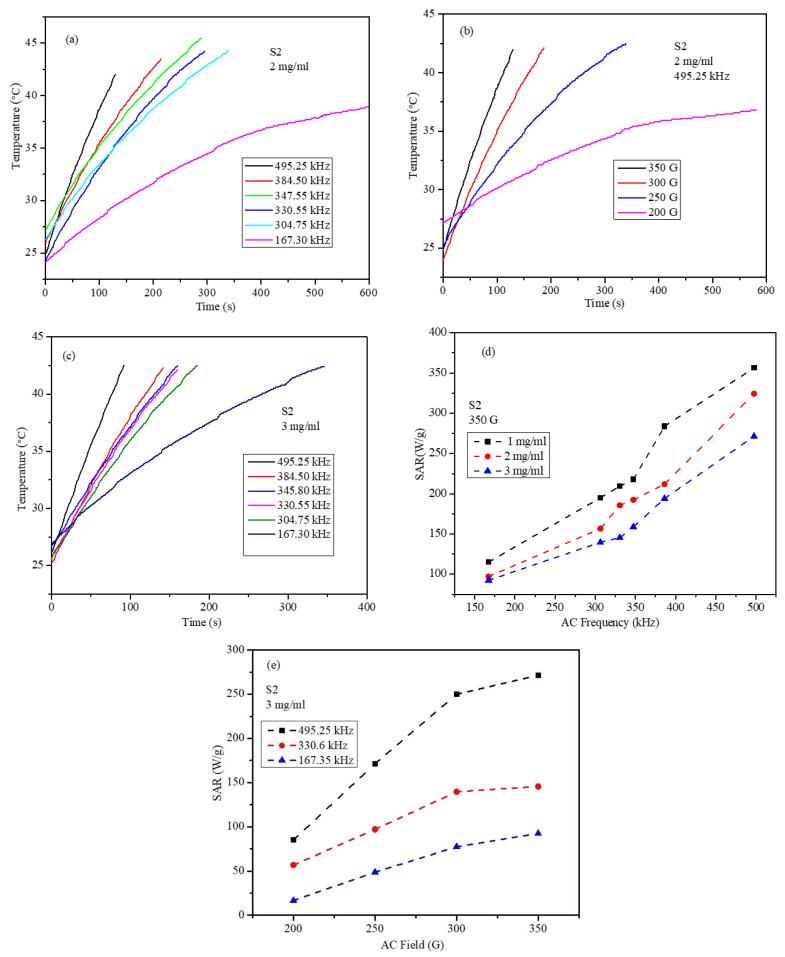
Heating profiles of the S2 core-shell nanoparticles; (**a**) frequency-dependent heating profiles of the S2 (2 mg/mL) nanoparticles under 350 G, (**b**) field-dependent heating profiles of the S2 (2 mg/mL) nanoparticles at a frequency of 495.25 kHz, (**c**) frequency-dependent heating profiles of the S2 (3 mg/mL) nanoparticles under 350 G, (**d**) concentration vs. frequency-dependent SAR under a field amplitude of 350 G, and (**e**) field-dependent SAR for the 3 mg/mL concentration at frequencies of 495.25, 330.6, and 167.35 kHz.

The heating profiles of the S2 nanoparticles for the concentrations of the 2 and 3 mg/mL ferrogels are shown in Figure 11a–c. The heating profiles show that the magnetic thermal conversion efficiency of the S2 nanoparticles depends on the concentration of the particles, field, and frequency of the AMF, as shown in Figure 11. The concentration-dependent magnetic heating of the nanoparticles is essential to study, as the nanoparticles are distributed randomly inside the tumor after administration, and different heating points would be created, such that few points may cross the threshold temperature of 50 °C. To address this issue, the treatment dosage should be selected based on phantom studies. The effect of the field frequency and field strength-dependent SAR values are shown in Figure 11d,e. As can be seen in Figure 11d, the SAR values of the S2 sample increase almost linearly with the increase in the AMF frequency for all concentrations, which roughly agrees with the expectations of the LRT as displayed in Equation (1). Figure 11e displays the SAR of the S2 sample at the highest concentration of 3 mg/mL, where an almost linear dependence of the SAR on the AMF amplitude is observed. Clearly, this behavior does not agree with the predictions of the LRT, where the SAR depends quadratically on the field amplitude.

**Figure 12:**
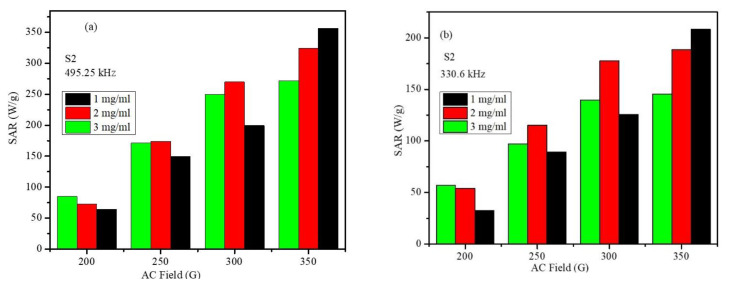
Concentration-dependent SAR values vs. the field at frequencies of (**a**) 495.10 kHz and (**b**) 330.6 kHz.

Figure 12a,b displays the SAR of the S2 core-shell nanoparticles at three different concentrations versus the applied field amplitude at frequencies of 495.10 kHz and 330.6 kHz. It can be seen that at the particle concentrations of 2 and 3 mg/mL, the SAR values have a nearly linear dependence on the applied field amplitude (at both frequencies), which clearly deviates from the quadratic behavior predicted with the LRT. However, for the low particle concentration of 1 mg/mL, the SAR values have nearly a quadratic dependence on the applied field amplitude (at both frequencies), as predicted with the LRT. As the LRT was derived for isolated (non-interacting) magnetic particles with a uniform size distribution, this behavior could be explained by understanding the role of dipolar interactions among the magnetic nanoparticles [41,42,43]. It is known that magnetic dipolar interactions between magnetic nanoparticles increase with a decrease in the average distance between the nanoparticles. Dipolar interactions affect the heating efficiencies of magnetic nanoparticles by influencing their magnetic relaxations. However, the role of dipolar interactions on the heating efficiency of magnetic nanoparticles is contradictory and not completely agreed upon [41,43]. In addition, dipolar interactions enhance effective anisotropy. With the increase in nanoparticle concentration (2 mg/mL and 3 mg/mL), the distances between the nanoparticles decrease, and the dipolar interactions become stronger, thus, deviations from the predictions of the LRT are expected. For the smallest particle concentration of 1 mg/mL, the distances between the particles are large, and the dipolar interactions become weak, thus, the SAR values are expected to agree with the predictions of the LRT.

### 2.5. Cytotoxicity Assay

**Figure 13:**
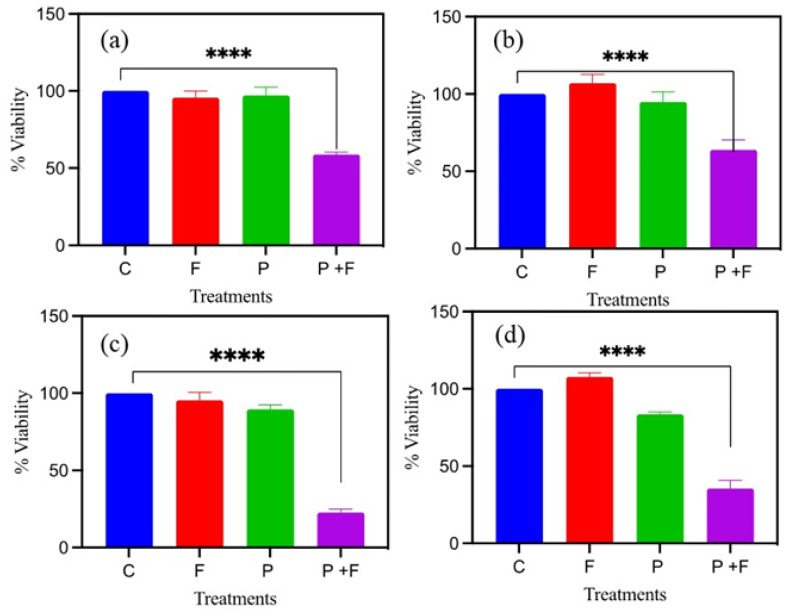
MTT assay after the AMF treatment; (**a**) MDA-MB-231 0 h, (**b**) HT-29 0 h, (**c**) MDA-MB-231 24 h, and (**d**) HT-29 24 h (**** indicates that P value is less than 0.0001).

The cytotoxicity, using 1 mg/mL of chitosan-coated S2 nanoparticles treated with an AMF (384.5 kHz and 350 G), decreased the viability of the MDA-MB-231 cells by approximately 42 and 61% after 0 and 24 h, respectively (Figure 13a,c). On the other hand, 71% of the HT-29 cells remained viable after the treatment with the AMF at 0 h. Then, the cell viability decreased to 41% after 24 h of treatment (Figure 13b,d). The results for the cell proliferation assay for the cells treated with the AMF showed a significant difference with the control group at *p* < 0.0001 for both cell lines at different time points. The viability of the cancer cells treated with the field alone was similar to that of the controls at different time points, indicating a lack of toxicity from the magnetic field (Figure 13a–d). The cells treated with the S2 particles alone showed slight toxicity only after 24 h, which could be due to the mechanical pressure of the particles cultured on top of the cells in the culture dishes. To study the effect of AMF exposure time, the cells were exposed to an AMF ((384.5 kHz and 350 G) for 30 min, and the cells showed higher viability compared to the 45 min exposure (supplementary data, Figure S3a,b). MDA-MB-231 cells were also treated with AMF 167.40 kHz and 780 G field strength for 45 min to study the effect of lower frequency and high AC field. 30% cells were dead after AMF exposure, no apoptosis was noted with groups treated with the field (F) and particle (P) alone (supplementary data, Figure S4a,b). The cell death is lower for 167.40 kHz and 780 G compared to the 384.5 kHz and 350 G AMF.

### 2.6. Apoptosis Assay

**Figure 14:**
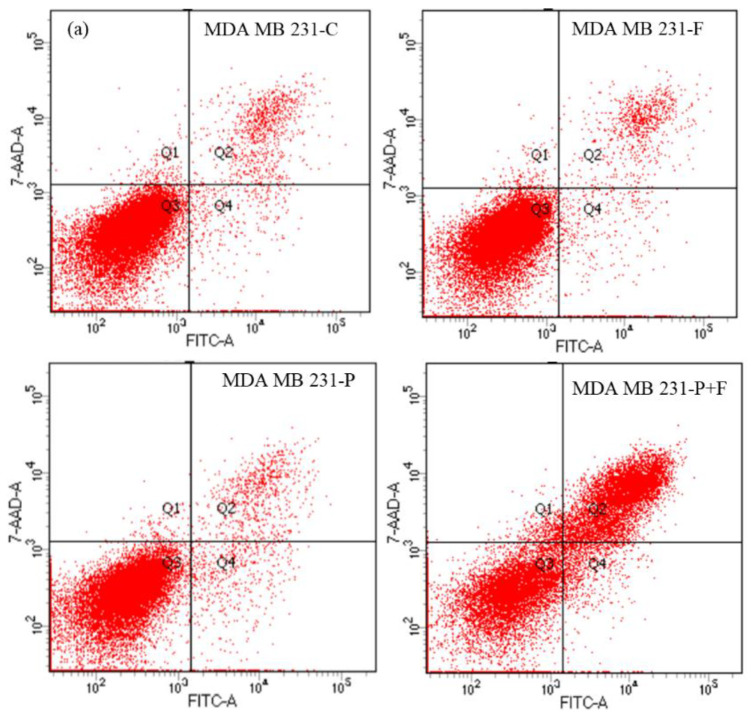

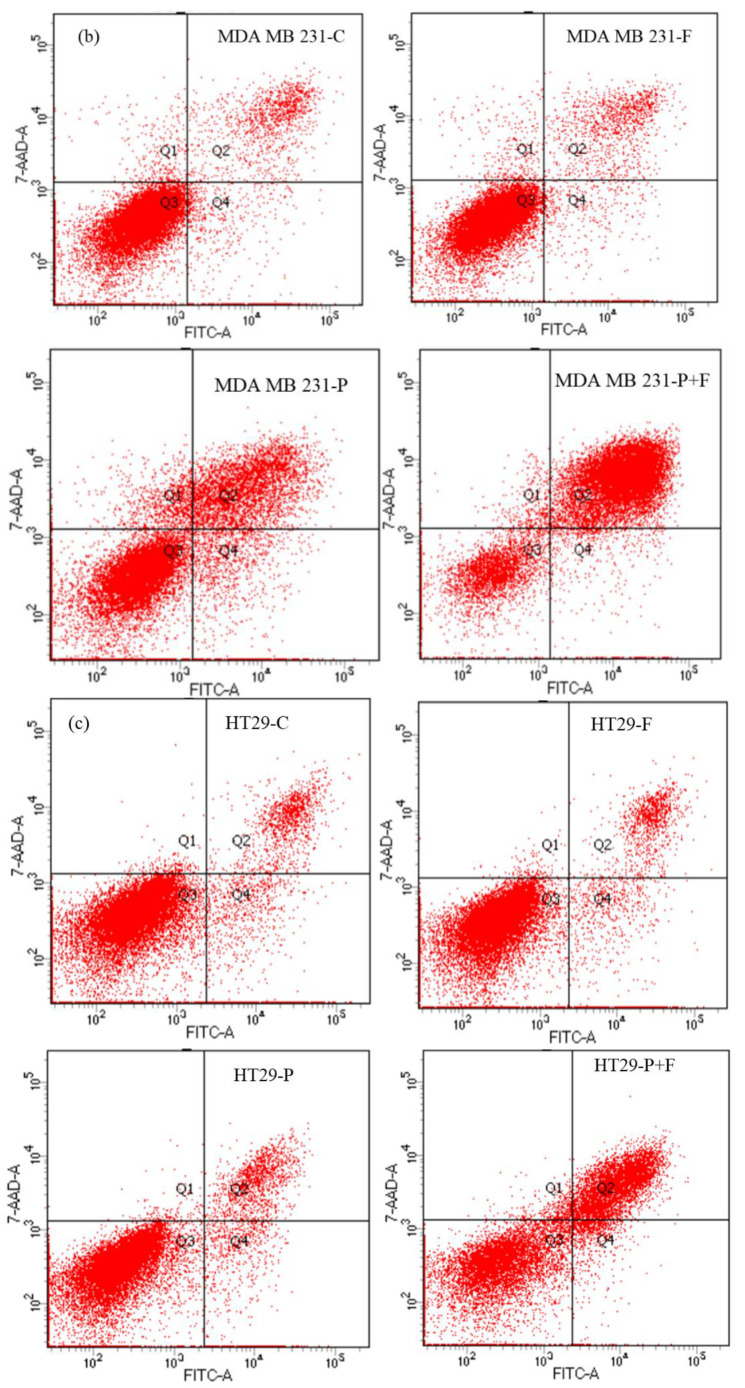

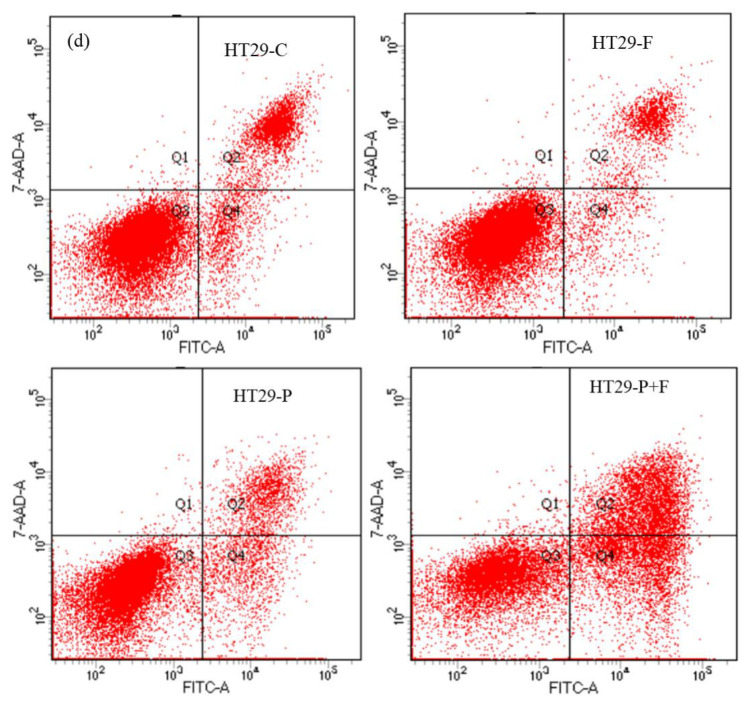
(**a**) Apoptosis assay of the MDA-MB-231 cell line 0 h after the AMF treatment. (**b**) Apoptosis assay of the MDA-MB-231 cell line 24 h after the AMF treatment. (**c**) Apoptosis assay of the HT29 cell line 0 h after the AMF treatment. (**d**) Apoptosis assay of the HT29 cell line 24 h after the AMF treatment.

Annexin V (or Annexin A5) is a member of the annexin family of intracellular proteins that bind to calcium-dependent phosphatidylserine (PS). In healthy cells, PS is generally restricted to the internal leaflet of the plasma membrane. However, during early apoptosis, membrane asymmetry is lost, and PS translocates to the exterior leaflet. Annexin V, tagged with a fluorochrome, is used to target and detect apoptotic cells selectively. Annexin V binding buffer is used for Annexin V staining. Annexin V binding cannot distinguish between apoptotic and necrotic cells on its own. 7AAD labeling differentiates between apoptosis and necrosis based on the differences in 7AAD permeability across live and damaged cell membranes. Due to the entry of these dyes into the nucleus, where they bind to DNA, early apoptotic cells reject 7-AAD, but late apoptotic cells stain favorably. 7-AAD (7-amino-actinomycin D) has a high DNA binding constant and is effectively rejected from intact cells. During flow cytometric analysis, when it is stimulated with laser light of a wavelength of 488, 7-AAD fluorescence is seen in the spectrum’s far-red region (650 nm long-pass filter). Following each AMF treatment, the MDA-MB-231 and HT-29 cells were examined for apoptosis (Figure 14a–d). The apoptotic rate of the MDA-MB-231 cells treated with the S2 magnetic nanoparticles and AMF for 0 h was 42.1%. This increased to 76.6% after 24 h (Figure 14b). Similarly, the apoptotic rate of the HT-29 cells increased over time from 41.8% to 58.1% after 0 and 24 h of treatment, respectively. Interestingly, no apoptosis was noticed when the groups treated with the field (F) and particle (P) alone were observed at any time point, except for the cells treated with particles (P) alone, which exhibited a low percentage of apoptosis. These results are in good correlation with those obtained from the cell viability assay and explain the level of cytotoxicity observed in the cancer cell lines after each treatment.

The cell death due to the thermal treatment is attributed to the protein denaturation and subsequent activation and deactivation of several downstream pathways. Protein denaturation begins at approximately 40 °C, and higher temperatures denature a larger percentage of proteins. Heat-induced denaturation and co-aggregation induce downstream processes leading to cell death, including protein synthesis inactivation, cell cycle advancement, and DNA repair. In addition, hyperthermia negatively affects the cytoskeleton, organelles, and intracellular transport. These heat-induced modifications in the plasma and subcellular organelle membranes, as well as membrane proteins, possibly contribute to a decrease in cell viability. The production of reactive oxygen species (ROS) from thermal shock induces apoptosis. Hyperthermia induces endoplasmic reticulum (ER) triggered apoptosis in multiple malignancies, including breast cancer, melanoma, skin cancer, colon cancer, and lung cancer [44,45]. Protein modifications, folding, synthesis, and lipid synthesis are all controlled by the ER [46]. When cells are subjected to numerous stimuli, such as oxidation, heat, medication, injury, or infection, ER homeostasis is interrupted and unfolded. Several signaling mechanisms, such as the unfolded protein response (UPR) or ER-associated protein degradation, are activated by the cells [47]. These responses protect cells, but extreme ER stress ultimately induces apoptosis. The ER chaperone proteins, glucose-related protein 78 (GRP78)/Bip and GRP94, are important markers and regulators of ER stress [48]. The GRP78 protein has anti-apoptotic characteristics and inhibits the UPR. Moreover, heat causes reactive oxygen species (ROS) and mitochondrial dysfunction in several cancer cell lines, showing that both factors play crucial roles in the apoptotic process.

## 3. Materials and Methods

### 3.1. Synthesis of the MnFe_2_O_4_ Nanoparticle

The nanoparticles were synthesized via the organometallic decomposition of metal-organic salts in high-boiling solvents [24]. To synthesize the MnFe_2_O_4_ nanoparticles, Mn(acac)2 (1 mmol), Fe(acac)3 (2 mmol), 1,2-hexadecanediol (5 mmol), oleic acid (6 mmol), oleylamine (6 mmol), and diphenyl ether (20 mL) were mixed in a three-neck round-bottom flask. The reaction mixture was heated at 150 °C for 30 min and finally heated to 255 °C for 30 min under a continuous argon flow. The reaction mixture was stirred throughout the reaction at 300 rpm, and the condenser fitted to the round-bottom flask was cooled using water flow. The nanoparticles synthesized were cooled to room temperature, 50 mL ethanol was added, and the nanoparticles were separated using an external magnet. The particles were washed using a mixture of ethanol and hexane using a centrifuge at 8000 rpm.

### 3.2. Synthesis of the Core-Shell Nanoparticles

To synthesize the MnFe_2_O_4_-Fe_3_O_4_ core-shell nanoparticles, MnFe_2_O_4_ nanoparticles as seeds were synthesized in the first step. The measured quantities (40 and 60 mg) of the seed nanoparticles were dispersed in 50 mL of hexane by sonication for 30 min. Into this solution, benzyl ether (20 mL), Fe(acac)_3_ (3 mmol), oleic acid (6 mmol), and oleylamine (6 mol) were added, and the solution mixture was heated at 100 °C for 30 min to evaporate the hexane. To this reaction mixture, 400 mg of 1,2-hexadecanediol was added, and the reaction mixture was heated for 30 min at 150 °C under an argon flow to dissolve the initial precursors. This reaction mixture was finally heated to 295 °C for 90 min and cooled to room temperature. 50 mL of ethanol was added to this reaction mixture, and the nanoparticles were separated using an external magnet and further washed using a mixture of ethanol and hexane by centrifugation (8000 rpm, 10 min).

### 3.3. Characterization of the Nanoparticles

The structural phases and average crystalline sizes of the nanoparticles were determined from the X-ray diffraction pattern using a Shimadzu-6100 powder XRD diffractometer fitted with the Cu-Kα radiation (wavelength 1.542 Å). The XRD patterns were recorded in the 20 to 70° 2θ range. A Titan Themis 300 kV from FEI transmission electron microscope (TEM) was used to obtain the bright-field images and selected area electron diffraction patterns. The nanoparticle size distributions were obtained using ImageJ software. The dc magnetic measurements were carried out using a VSM in the Physical Properties Measurement System (PPMS) from Quantum Design.

### 3.4. Cell Culture

The MDA-MB-231 human breast cancer cell line (ATCC^®^ HTB-26, ATCC, Manassas, VA, USA) and HT-29 cells human colorectal cancer (Addexbio/San Diego, CA, USA) cells were cultured in Roswell Park Memorial Institute (RPMI) and Mccoy’s media medium (Sigma-Aldrich Co., St. Louis, MI, USA), supplemented with 10% FBS and 1% penicillin/streptomycin, respectively. All cell lines were incubated under humidified air and 5% CO_2_ at 37 °C for further studies.

### 3.5. Cytotoxicity Assay

The MDA-MB-231 and HT-29 cell lines were seeded at a density of 5000 cells/well in triplicates on a 96-well tissue culture plate (Corning, Sigma-Aldrich Co., St. Louis, MO, USA). The MTT [3-(4, 5-dimethylthiazol-2-yl)-2, 5-diphenyltetrazolium bromide] test was used to determine the cytotoxicity of the S2 magnetic nanoparticle treated with an AMF with a frequency of 384.50 kHz and a 350 G field strength. The control group (C) cells were maintained at 37 °C in the incubator throughout the experiment. One set of cells (F) was treated with a magnetic field without nanoparticles to examine the effect of the magnetic field. One more group of cells (P) was treated with nanoparticles without a field to study their effect alone. For the AMF treatment, the cells (P + F) were treated with 1 mg/mL of S2 nanoparticles. The cell viability was measured at 0 and 24 h post-AMF treatment. At specified time intervals, the cells were washed three times with phosphate-buffered saline (PBS) to remove the nanoparticles from the cell surface, followed by the addition of 100 µL of culture medium and 10 µL of MTT solution (5 mg/mL in PBS) and incubation for 2 h under humidified air and 5% CO_2_ at 37 °C. The MTT-containing medium was then removed, and formazan crystals were dissolved in 100 µL of dimethyl sulfoxide. The absorbance of the MTT assay was measured at 570 nm using an ELISA plate reader Synergy^TM^ HTX microplate reader (BioTek, Winooski, VT, USA). The cell viability percentage was calculated by using the following equation:

(4) $$\mathrm{Cell}\;\mathrm{viability}\left(\%\right)=\frac{\mathrm{OD}\;\mathrm{of}\;\mathrm{test}\;\mathrm{well}}{\mathrm{OD}\;\mathrm{of}\;\mathrm{reference}\;\mathrm{well}}\;\times 100$$

### 3.6. Apoptosis Assay

The apoptotic and necrotic cell deaths were studied using MDA-MB-231 and HT-29 cells after the treatment with the AMF. The analysis of cell viability and the presence of apoptotic markers were conducted to determine the impact of the AMF treatment on the cancer cells. Similar to the MTT assay, four groups (C, F, P, and P + F) of MDA-MB-231 and HT-29 cells were treated with an AMF, and a flow cytometry test was carried out at 0 and 24 h. The AMF-treated cells, along with the controls, were cultured in 5% CO_2_ at 37 °C for 24 h. At each time point, the cells were washed with PBS and transferred to a 15-mL centrifuge tube containing media. The cells were then resuspended in 500 μL of bind Buffer, followed by the addition of 5 μL of Annexin V-FITC and 5 μL of PI. After 15 min, the flow cytometric analysis was conducted using BD Biosciences, San Jose, CA, USA.

### 3.7. Magnetothermal Measurements

The agar ferrogels of the nanoparticles were prepared with known concentrations of nanoparticles to obtain the homogeneously distributed particle gel. The heating profiles of the nanoparticles were obtained using the nanoScale Bio magnets hyperthermia instrument. The magnetothermal measurements were conducted using an AMF with an amplitude of 350 G and frequencies of 495.25, 384.50, 345.80, 330.55, 304.75, and 167.30 kHz. The increase in the temperature was measured with respect to time. The SAR values are provided with Equation (3) [23]:

(5) $$\mathrm{SAR}\;\left(W/g\right)=\frac{C}{m_{\mathrm{MNP}}}\frac{\mathrm{dT}}{\mathrm{dt}}$$

where C (in J/K) is the heat capacity of the ferrogel, which is the sum of the specific heat capacities multiplied by the mass of the components (MNPs, water, and agar), $m_{\mathrm{MNP}}$ is the mass (in g) of the MNPs in the ferrogel, and $\frac{\mathrm{dT}}{\mathrm{dt}}$ is the initial slope of the temperature-time curve. While calorimetric measurements were used to determine the SAR, adiabatic conditions are preferred where external heat transfer is minimized. However, it is not an easy task to build adiabatic measurement systems. In addition, the measurements in such systems would be time-consuming. Hence, SAR measurements are usually performed in non-adiabatic systems. In such cases, the results might not be very accurate. However, in our study, the SAR values were obtained from the initial slope of the temperature-time curve in the first 20–40 s. Hence, the heat transfer between the sample and the environment was minimized. In addition, in the initial heating process, it is expected that the temperature variations within the sample would be very small, and the adiabatic conditions are considered valid [49].

## 4. Conclusions

MnFe_2_O_4_-Fe_3_O_4_ core-shell nanoparticles were synthesized using an organometallic decomposition method, and the magnetic properties were studied in the temperature range of 5–300 K. Two sets of nanoparticles with average sizes of 9.1 ± 2.2 (S1) and 10.7 ± 1.3 (S2) nm were studied. The core size (5.0 ± 0.5 nm) was fixed in both particles with shell thicknesses of 4.1 and 5.7 nm for the S1 and S2 nanoparticles, respectively. The exchange bias displayed significant non-monotonic temperature and field dependencies in both samples. The observed temperature-dependent exchange bias properties are attributed to the existence of spin glass (SG) like regions at the core-shell interface due to interface spin freezing. The effective anisotropy constant was found to decrease non-linearly with temperature in both samples, but the magnitude of the S2 sample was slightly higher than that of the S1 sample at all temperatures. We attribute this behavior to the larger interface SG regions in the S2 sample, which provided indirect exchange coupling between the core and shell phases. The SAR values for both core-shell NPs displayed much larger values than those for the MnFe_2_O_4_ seed NPs, indicating a significant enhancement due to the core-shell structure. However, the SAR values for the S2 sample were higher than those of the S1 sample under the same field parameters. The in vitro treatment of the human breast cancer cell line, MDA-MB-231, and the human colorectal cancer cell line, HT-29, were conducted at selected frequencies and field strengths to evaluate the efficiency of the S2 nanoparticle to kill the cancer cells. The cellular cytotoxicity was estimated using flow cytometry and an MTT assay at 0 and 24 h after the treatment with an AMF. The cells subjected to 45 min of the AMF (384.50 kHz and 350 G) showed a 70% decrease in cell viability.

## Figures and Tables

**Table 1:** The list of lattice constants for the core-shell and individual phases of the nanoparticles.

Nanoparticles	Particle Sizes (nm)	Lattice Constant (Å)
MnFe_2_O_4_	5.4	8.4297
S1-core-shell	9.6	8.3844
S2-core-shell	11.7	8.4010

**Table 2:** The (311) peak positions obtained for the individual MnFe_2_O_4_ and Fe_3_O_4_ nanoparticles and core-shell nanoparticles.

Particles	(311) Peak Position
Fe_3_O_4_	35.5723 ± 0.0014
MnFe_2_O_4_	34.9869 ± 0.0001
Mixture	35.0461 ± 0.0019
S1-core-shell	35.4629 ± 0.0037
S2-core-shell	35.4029 ± 0.0019

**Table 3:** List of the average sizes, effective anisotropy, and SAR values of the MnFe_2_O_4_ seed and core-shell nanoparticles.

Compositions	Average Sizes from the XRD (nm)	Average TEM Sizes (nm)	Shell Thickness (nm)	M_S_ (emu/g)	K_eff_ 10^3^ J/m^3^	SAR (W/g) at 495.25 kHz and 350 G
MnFe_2_O_4_ seeds	5.4	5.0 ± 1.2		39.07		150.6
S1 [Fe_3_O_4_(MnFe_2_O_4_)]	9.5	9.1 ± 2.2	4.1	70.40	1.946	311.8
S2 [Fe_3_O_4_(MnFe_2_O_4_)]	11.7	10.7 ± 1.3	5.7	69.90	2.216	356.5

## Data Availability

Not applicable.

## Supplementary Materials

The following supporting information can be downloaded at: https://www.mdpi.com/article/10.3390/ijms232314825/s1. References [50,51,52,53] are cited in the Supplementary Material.
