# Supplementary material for: Hyperthermia of Magnetically Soft-Soft Core-Shell Ferrite Nanoparticles
Source: Int J Mol Sci. 2022 Nov 26;23(23):14825. doi: 10.3390/ijms232314825 (PMC9735482; doi:10.3390/ijms232314825)
Supplement: Supplementary file 1 [file ijms-23-14825-s001.zip › ijms-1988989-supplementary.pdf]

Supplementary Data

1. *Effect of Super paramagnetic Properties on Magnetothermal ability of the nanoparticles*

The heating of superparamagnetic MNPs in small amplitude AMF (such as the Zeeman energy, which is smaller than the thermal energy) is represented by equation 1. According to Rosensweig's model, the magnetization of nanoparticles is proportional to the applied magnetic field, with the proportionality element being the complex susceptibility. In an AMF, the rate of volumetric heat release is given by the equation (S1) [1].

$$P = \pi\mu_0\chi'' H^2 f \tag{S1}$$

μ_0 ------vacuum magnetic permeability

f ------ frequency

H------ amplitude of the AMF

χ''----- imaginary part of the magnetic susceptibility ($\chi = \chi' - i\chi''$).

In the LRT, it is assumed that χ stays constant as H increases $M = \chi H$. In the LRT, the heat dissipation of the MNPs has a linear dependence on the AMF frequency and a quadratic dependence on AMF amplitude. The imaginary part of the susceptibility, χ" is given by equation (S2) [2]:

$$\chi'' = \frac{2\pi f\tau}{1 + (2\pi f)^2}\chi_0 \tag{S2}$$

The static susceptibility χ_0 is given by

$$\chi_0 = \frac{\mu_0 M_s^2 V}{k_B T} \tag{S3}$$

Ms is the saturation magnetization of the material, V its magnetic volume, k_B is the Boltzmann constant and T is the absolute temperature.

The effective magnetic relaxation time τ is given by

$$\frac{1}{\tau} = \frac{1}{\tau_N} + \frac{1}{\tau_B} \quad \text{(S4)}$$

The Brownian relaxation time, τ_B characterizes particle's magnetic moment flipping due to the rotation of the particle itself, and is given by:

$$\tau_B = \frac{3\, V_H\, \eta}{k_B\, T} \quad \text{(S5)}$$

Where V_H is the hydrodynamic volume of the particle, and η is the viscosity of the liquid where the particle is immersed. Since the Brownian relaxation time as stated in the equation (S5) depends on the viscosity of the surrounding medium the effect becomes more pronounced when heating ability is obtained for ferrogel.

The Neel relaxation time, τ_N is due to the rotation of the magnetic moment of the MNP, and is given by [3]:

$$\tau_N = \frac{\tau_o}{2}\sqrt{\frac{\pi k_B T}{KV}}\, exp\left(\frac{KV}{k_B T}\right) \quad \text{(S6)}$$

Where K is the magnetic anisotropy of the MNPs and τ_0 is a constant $\approx 10^{-13} - 10^{-9} s$. V is the volume of the magnetic core of the particle.

2. Average crystalline size calculations using Scherrer method

The average sizes of the nanoparticles presented in this study are determined using Scherrer method. The FWHM of the highest intensity peak (311) is determined using the gaussian fitting in origin software. The average sizes of the nanoparticles are calculated using Scherrer equation (S7)[4]:

$$D = \frac{k\lambda}{\beta \cos\theta} \quad \text{(S7)}$$

where D is the crystalline size of the particle, K is the Scherer's constant (K=0.94), λ is the X-ray wavelength (1.5417Å), β is full width at half maximum (FWHM) of the (311) diffraction peak.

3. The MH plots of the nanoparticles under field cooling 0,1,2, and 3 T

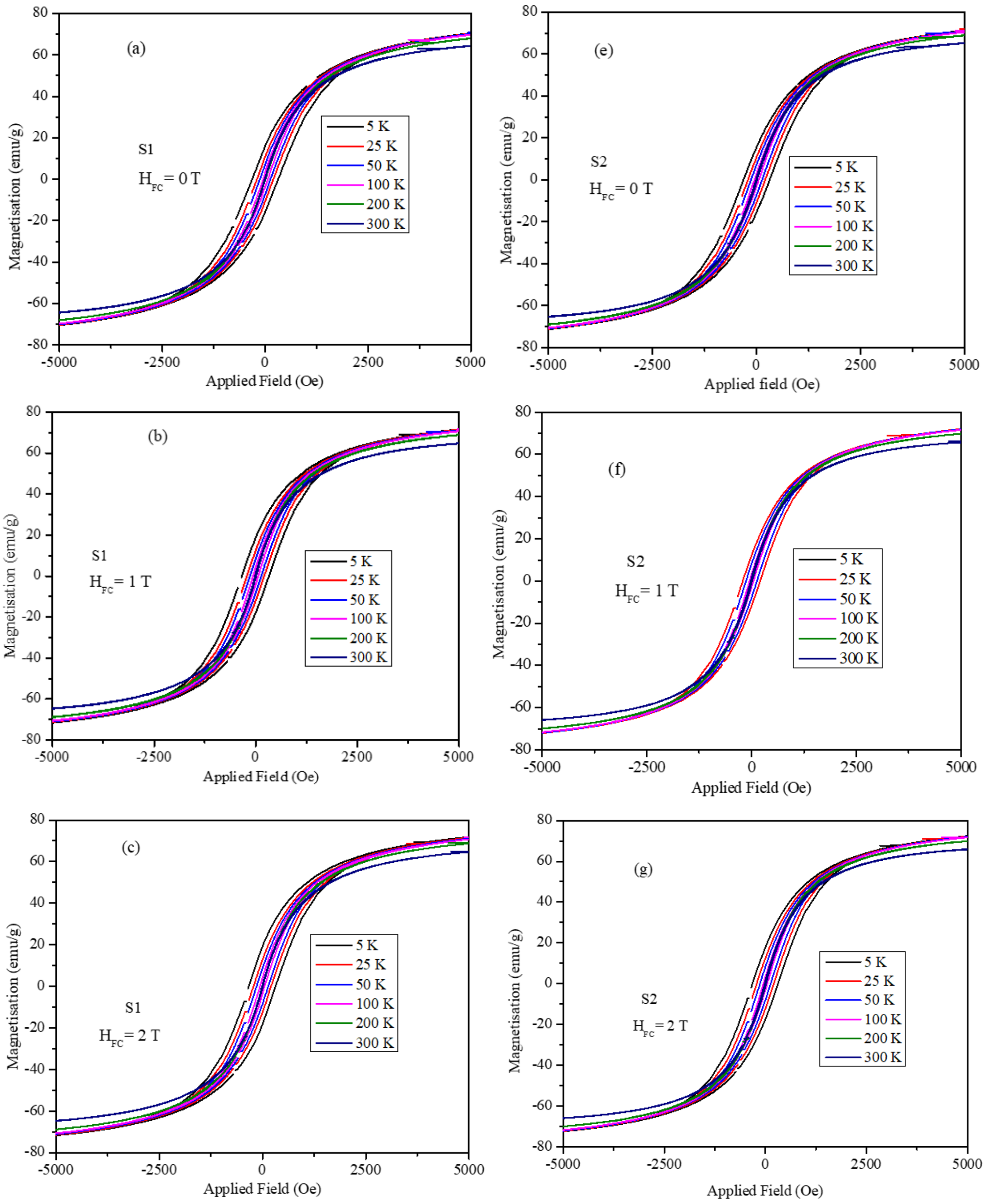

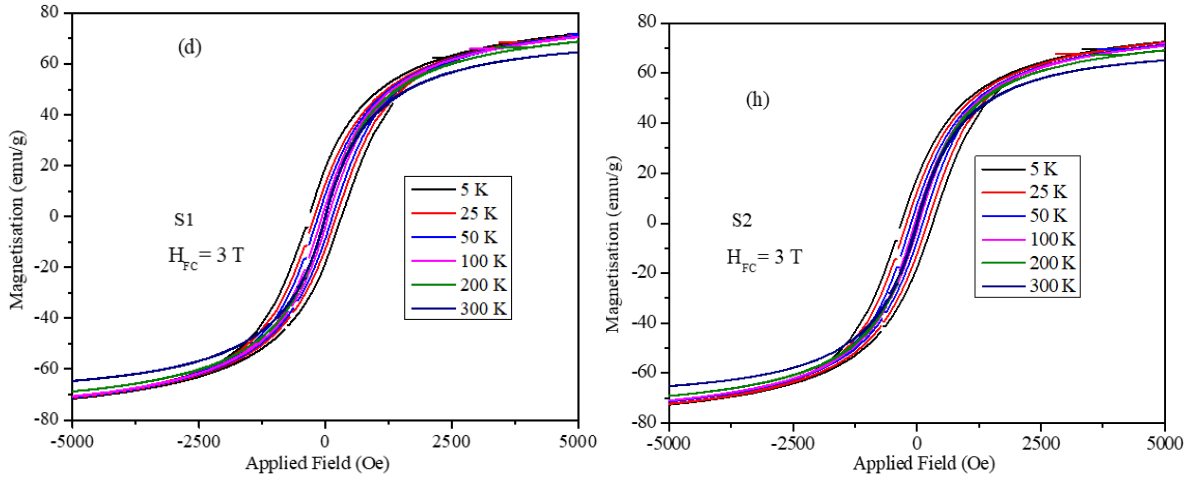

Figure S1. Magnetic hysteresis loops obtained at room temperature in the field range of -5000 Oe to +5000 Oe obtained under the cooling of 0,1,2 and 3 T magnetic field for (a-d) S1 and (e-h) S2.

4. Calculation of H_{EB}, H_C, and M_Y

The low temperature MH plots obtained in the temperature range of 5-300 K under 0,1,2, and 3 T field cooling are shown in Figure 1(a-h). The exchange bias values of the core-shell nanoparticles were obtained from these hysteresis loops. The horizontal shift in the hysteresis loops was defined as the exchange bias field, H_{EB}. The exchange bias field, H_{EB} was calculated using the equation (S8):

$$H_{EB} = \frac{H_{C1} + H_{C2}}{2} \qquad \text{(S8)}$$

Here the coercive field at the descending branch of the hysteresis loop is H_{C1}, and the ascending branch is H_{C2}.

The vertical shift in the hysteresis loops was calculated using the following formula:

$$M_y = \frac{M_{R1} + M_{R2}}{2} \qquad \text{(S9)}$$

Here the remnant magnetization value at the descending branch of the hysteresis loop is M_{R1} and the ascending branch is M_{R2}.

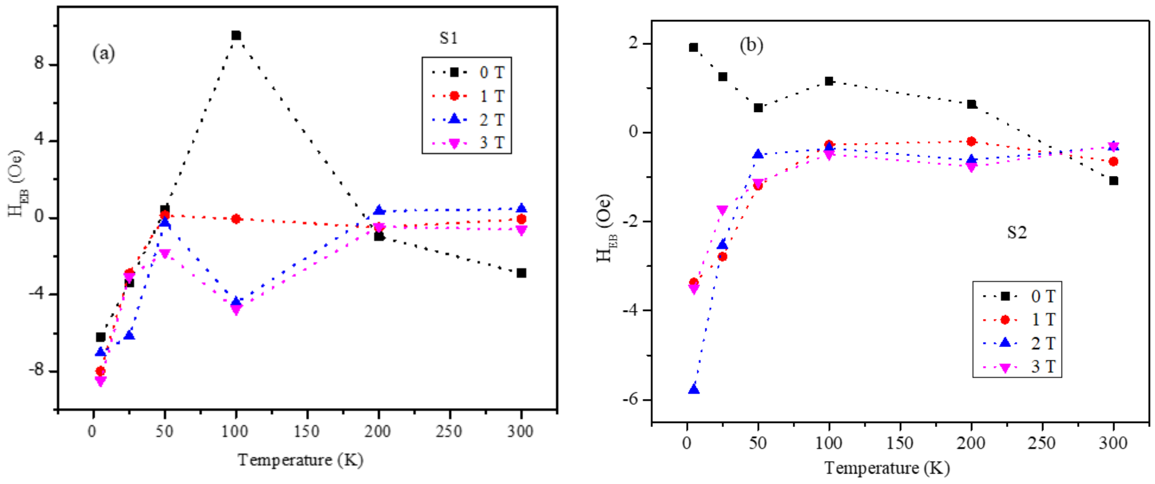

Figure S2. The temperature dependence of H_{EB} in the temperature range of 5–300 K for (a) S1 (b) S2.

5. Treatment of MDA-MB-231 cells with AMF

The cytotoxicity of 1 mg/ml chitosan-coated nanoparticles treated with AMF (384.5 kHz and 350 G) field strength for 30 minutes lowered the viability of MDA-MB-231 cells by approximately 30%. The results of the cell proliferation experiment for cells treated with AMF differed significantly from those of the control group. The viability of cancer cells treated with field alone was comparable to that of the control, demonstrating any toxicity from field alone. It has been demonstrated in vitro that the temperature and duration of AMF therapy have a significant effect on cell survival.

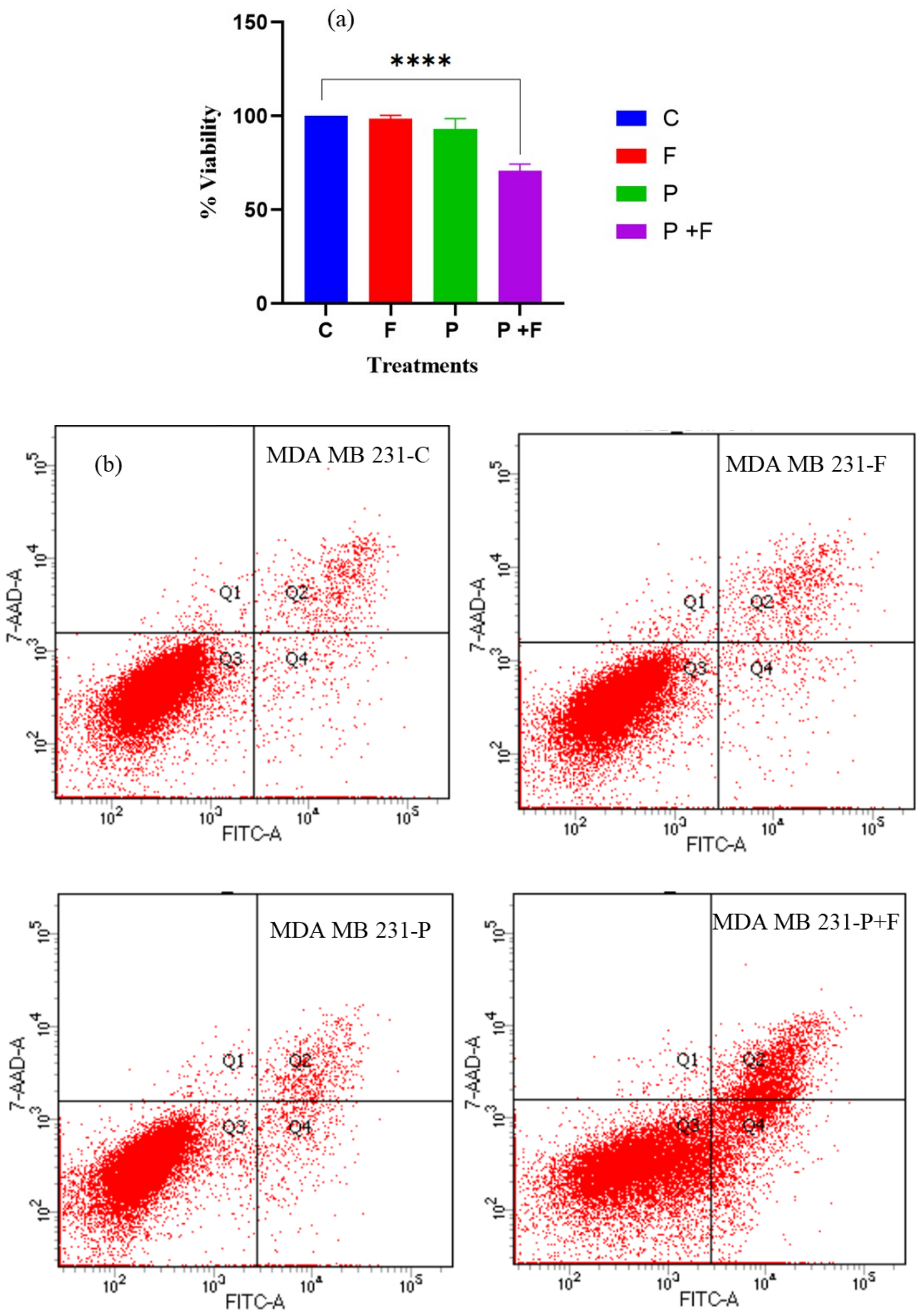

Figure S3. (a) MTT assay of MDA-MB-231, (b) Apoptosis assay of MDA-MB-231 treated with AMF 384.5 kHz and 350 G field strength for 30 minutes.

MDA-MB-231 cells were also treated with AMF 167.40 kHz and 780 G field strength for 45 minutes to study the effect of lower frequency and high AC field. 30% cells were dead after AMF exposure, no apoptosis was noted with groups treated with the field (F) and particle (P) alone. The cell death is lower (167.40 kHz and 780 G) compared to the 384.5 kHz and 350 G AMF.

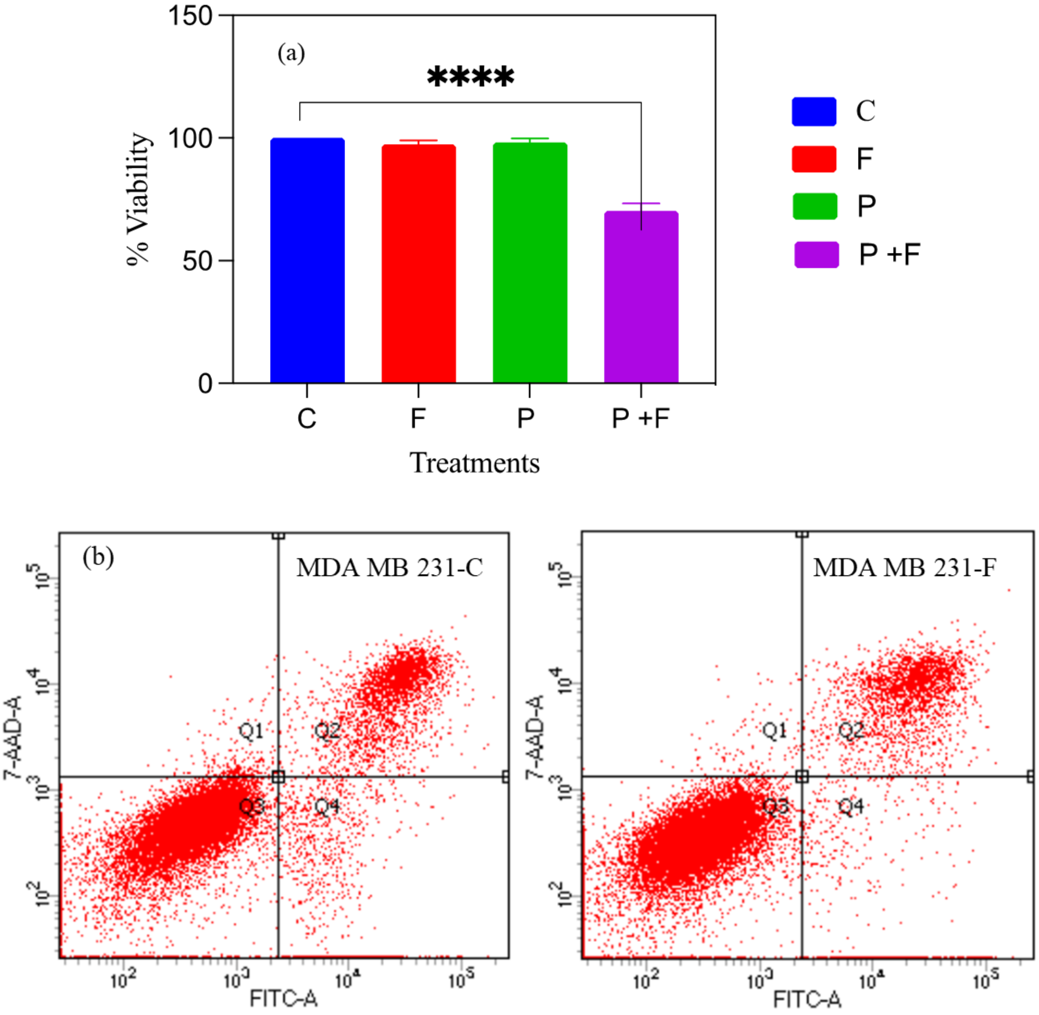

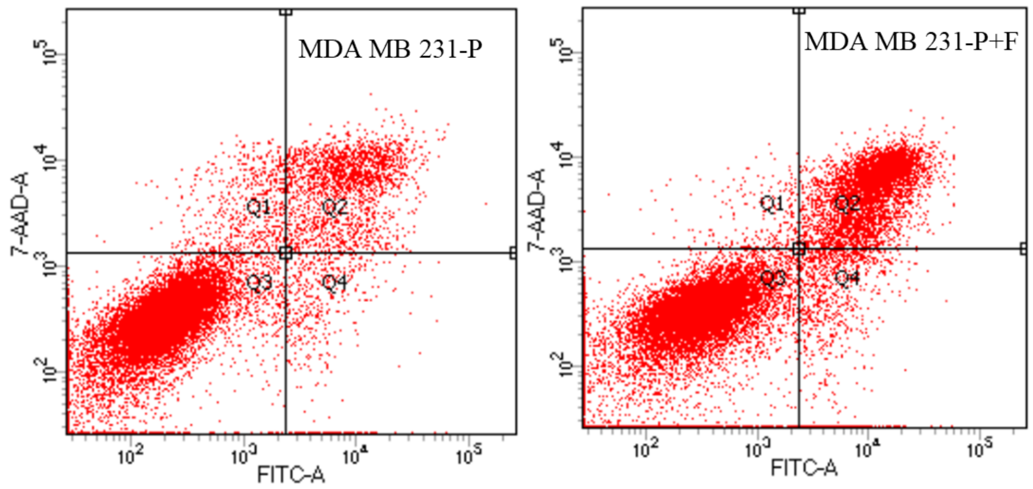

Figure S4. (a) MTT assay of MDA-MB-231, (b) Apoptosis assay of MDA-MB-231 treated with AMF 167.40 kHz and 780 G field strength for 45 minutes.

References

1. Rosensweig, R.E. Heating Magnetic Fluid with Alternating Magnetic Field. *Journal of Magnetism and Magnetic Materials* **2002**, *252*, 370–374, doi:10.1016/S0304-8853(02)00706-0.
2. Glöckl, G.; Hergt, R.; Zeisberger, M.; Dutz, S.; Nagel, S.; Weitschies, W. The Effect of Field Parameters, Nanoparticle Properties and Immobilization on the Specific Heating Power in Magnetic Particle Hyperthermia. *J. Phys.: Condens. Matter* **2006**, *18*, S2935–S2949, doi:10.1088/0953-8984/18/38/S27.
3. Laurent, S.; Dutz, S.; Häfeli, U.O.; Mahmoudi, M. Magnetic Fluid Hyperthermia: Focus on Superparamagnetic Iron Oxide Nanoparticles. *Advances in Colloid and Interface Science* **2011**, *166*, 8–23, doi:10.1016/j.cis.2011.04.003.
4. Patterson, A.L. The Scherrer Formula for X-Ray Particle Size Determination. *Phys. Rev.* **1939**, *56*, 978–982, doi:10.1103/PhysRev.56.978.
